# Authors’ reply

**Published:** 2011

**Authors:** S Patyal, Ajay Banarji, Madhu Bhadauria, V S Gurunadh

**Affiliations:** Department of Ophtalmology, Armed Forces Medical College (AFMC), Pune, Maharashtra, India

Dear Editor,

I thank Chowdhury[1] for the interest in our case report.[2] The tumor protruded from the lateral canthus of the right eye and the left eye was within normal limits. The error is regretted.

Pleomorphic adenoma arising from an ectopic lacrimal gland is a rarity. The literature search did not yield any results of recurrent pleomorphic adenoma arising from an ectopic lacrimal gland.
